# Behçet's Uveitis

**DOI:** 10.4103/0974-9233.58425

**Published:** 2009

**Authors:** Ilknur Tugal-Tutkun

**Affiliations:** Department of Ophthalmology, Istanbul University, Istanbul Faculty of Medicine, Istanbul, Turkey

**Keywords:** Behçet's Disease, Immunomodulatory Treatment, Retinal Vasculitis, Uveitis

## Abstract

Behçet's disease is a multisystem inflammatory disorder that is most common in countries along the ancient “Silk Road”. The eye is the most commonly involved vital organ in Behçet's patients and the typical form of involvement is a relapsing remitting panuveitis and retinal vasculitis. Uveitis is the initial manifestation of the disease in 10-15% of the patients. Anterior uveitis is always nongranulomatous. Diffuse vitritis, retinal infiltrates, sheathing of predominantly retinal veins, and occlusive vasculitis are the typical signs of posterior segment inflammation. Spontaneous resolution of acute inflammatory signs is a diagnostic feature. Fundus fluorescein angiography is the gold standard in monitoring inflammatory activity. Laser flare photometry is a useful noninvasive tool since flare readings correlate with fluorescein angiographic leakage. The most common complications are cataract, maculopathy, and optic atrophy. Male patients have a more severe disease course and worse visual prognosis. Immunomodulatory therapy is indicated in all patients with posterior segment involvement. Corticosteroids combined with azathioprine and/or cyclosporine is used initially. Biologic agents, including interferon alfa and infliximab, are used in resistant cases. Visual prognosis has improved in recent years with an earlier and more aggressive use of immunomodulatory therapy and the use of biologic agents in resistant cases.

## INTRODUCTION

Behçet's disease is a multisystem inflammatory disorder of unknown etiology. It was named after the Turkish dermatologist, Professor Hulusi Behçet, who described the triple-symptom complex of the disease, recurrent oral ulcers, genital ulcers, and iritis, as a distinct entity in 1937.^1^ The disease is now recognized as a systemic vasculitis involving many organ systems and leading to a wide spectrum of manifestations.^2^

## EPIDEMIOLOGY

Although Behçet's disease occurs worldwide, it has the highest prevalence in countries between the northern latitudes of 30 and 45 degrees along the ancient “Silk Road” from the Mediterranean basin to the far East.^3^,^4^ The highest prevalence rates have been reported from Turkey (up to 420 per 100,000).^5^ The disease is strongly associated with the major histocompatibility complex antigen HLA-B51.^6^ The global distribution of this antigen among healthy control populations roughly corresponds to the overall distribution of the disease.^3^

## ETIOPATHOGENESIS

The etiopathogenesis of Behçet's disease has not been clarified. It is generally accepted that in immunogenetically susceptible individuals, environmental agents may trigger an enhanced and dysregulated immune response resulting in inflammatory vascular injury in many organ systems.^7^ A dysregulation of both innate and adaptive immune systems is implicated in the pathogenesis.^7^,^8^

## DEMOGRAPHICS

Behçet's disease primarily affects young adults. The age of onset of the disease is typically in the third or fourth decade of life. Onset of disease in childhood or at an advanced age is rare.^9^–^11^ Most pediatric cases are diagnosed in late childhood.^9^,^10^ Although both genders are equally affected in large series, male patients have a more severe course with more frequent involvement of vital organs.

## DIAGNOSIS

The diagnosis of Behçet's disease is based on a combination of clinical findings. There is no specific diagnostic test. Several sets of diagnostic criteria have been developed. The set of criteria proposed by the International Study Group for Behçet's Disease in 1990 was intended for classification of patients; to ensure uniformity of patients recruited for studies.^12^,^13^ Recurrent oral ulcers plus at least two of the following criteria are required for classification: Recurrent genital ulcers, skin lesions, eye lesions, or a positive pathergy test.

## SYSTEMIC MANIFESTATIONS

Patients with Behçet's disease have recurrent inflammatory attacks in all organ systems involved.^2^,^14^,^15^ Mucocutaneous manifestations are the hallmark of the disease. Recurrent painful oral ulcer, indistinguishable from common aphthae, is the earliest sign of the disease in majority of the patients. However, most patients do not seek medical attention until the development of other manifestations. Genital ulcers are painful and heal with scarring. A variety of skin lesions may be seen during the course of the disease, including erythema nodosum, superficial thrombophlebitis, papullopustular lesions, pseudofolliculitis, acneiform lesions, and rarely extragenital ulcerations. A hyperreactivity to nonspecific trauma is a pathognomonic feature of Behçet's disease. The skin pathergy reaction is defined by the development of papulopustular lesions at skin prick sites at 48 hours. Similar hyperreactivity can also be induced following trauma at other sites such as the joints, oral and genital mucosa, and conjunctiva. Arthralgias are common, but true arthritis develops in around 30% of the patients. The arthritis attacks are usually non-erosive, most commonly involving the knees and ankles. A chronic destructive arthritis is very rare. Gastrointestinal ulcers, neurological involvement, and major vessel disease are uncommon life-threatening complications of the disease.^16^ Gastrointestinal involvement is more common in the Japanese population than in the Mediterranean and the Middle Eastern populations. Ileocaecal region of the gastrointestinal tract is most frequently affected and may cause perforation. There are basically two types of neurological involvement: Parenchymal involvement which is mainly a meningoencephalitis most frequently affecting the brainstem structures and non-parenchymal involvement or vascular neuro-Behçet which represents mainly dural sinus thrombosis.^17^ Parenchymal neurological involvement has a worse prognosis with a higher rate of morbidity and mortality. Dural sinus thrombosis is more frequently seen in patients with vascular Behçet which is defined by deep venous thrombosis and arterial aneurysms and less commonly arterial occlusions.

## OCULAR INVOLVEMENT

The eye is the most commonly involved vital organ in Behçet's disease. Uveitis is reported in around 50% of the patients in multidisciplinary settings, but in more than 90% in ophthalmology departments.^14^,^18^ Uncommon or rare types of ocular involvement include episcleritis, scleritis, conjunctival ulcers, keratitis, orbital inflammation, isolated optic neuritis, and extraocular muscle palsies.^2^,^19^–^22^

## BEHÇ;ET'S UVEITIS

It is classically defined as a bilateral nongranulomatous panuveitis and retinal vasculitis. However, a minority of patients, especially females, may have isolated anterior uveitis. The disease may also remain unilateral for many years in some patients.^23^

The current diagnostic or classification criteria sets do not allow diagnosis of Behçet's uveitis based on ocular findings alone. Uveitis is the initial manifestation in around 10-15% of the patients with ocular involvement. Therefore, it is important to recognize Behçet's uveitis as a distinct entity that can be diagnosed in the absence of systemic manifestations. There are some pathognomonic findings that suggest the diagnosis at a single ocular examination in some patients. In most other cases, however, one needs to follow the patient and observe the characteristic clinical course.

Similar to the recurrent nature of the other manifestations of the disease, Behçet's uveitis runs a relapsing and remitting course. Sudden onset of uveitis attacks and spontaneous resolution of acute inflammatory signs are the rule and should be used as a diagnostic feature. Vision may be severely affected at the onset of explosive uveitis attacks but usually improves remarkably following the resolution of intraocular inflammation.

At a given episode of activation, acute inflammatory signs may be seen in the anterior or posterior segments of the eye or more commonly in both. Ciliary injection is not a constant feature in eyes with anterior segment inflammation. It may be disproportionately mild in eyes with severe anterior uveitis. A “cold” hypopyon resembling the pseudohypopyon, seen in masquerade syndromes, is not uncommon. Endothelial dusting is seen in eyes with a high grade of anterior chamber cells. Granulomatous keratic precipitates are not compatible with the diagnosis of Behçet's uveitis. A hypopyon is reported in 10–30% of the patients [Figure 1a]. Its incidence may be higher than the reported Figures because it may be missed if the patient is not seen at the onset of the uveitis attack. The hypopyon forms and dissolves rapidly. It typically forms a smooth layer and shifts freely with head positioning. In eyes with a hypopyon, there is almost always severe inflammation in the posterior segment. These features help differentiate Behçet hypopyon from HLA-B27 hypopyon, which is always “hot” and sticky, and with inflammation confined to the anterior segment.

Diffuse vitritis is a constant feature of posterior segment involvement. Vitreous haze is an indicator of inflammatory activity and is most severe at the onset of uveitis attacks. In severe attacks, the fundus reflex may be lost. If the fundus can be visualized, one may see hyperemia and swelling of the optic disc, diffuse inflammatory sheathing of the retinal veins, retinal infiltrates, branch retinal vein occlusions, and/or exudative retinal detachment. These acute inflammatory signs do not get worse, but gradually resolve even without treatment. Spontaneous resolution is an important diagnostic feature. However, new attacks may occur with new infiltrates or occlusive vasculitis of different branches, before the complete resolution of previous signs. Transient superficial retinal infiltrates that denote activation are considered as one of the pathognomonic findings [Figure 1b]. They may be in any number or location and resolve within a few days, usually without leaving any visible scarring. Deeper retinal infiltrates that may be sometimes difficult to distinguish from infectious retinal infiltrates may take longer to resolve and may leave scars. Another pathognomonic finding is the accumulation of inflammatory precipitates on the surface of the inferior peripheral retina during the resolution of diffuse vitritis [Figure 1c]. They appear several days after the onset of an attack and resolve within a few weeks without leaving any sequelae.

Periphlebitis, a hallmark of Behçet's vasculitis may be both leaky and occlusive. Although retinal arterioles and capillaries are also involved, arteriolitis is not seen without periphlebitis and capillaritis can be best demonstrated by fluorescein angiography. Sheathing of retinal veins might be difficult to visualize at the onset of an acute episode but may become readily visible when diffuse gliotic sheathing appears after the resolution of acute inflammation. More severe breakdown of the blood-retina barrier may cause a fundus picture resembling frosted-branch angiitis, also with hemorrhages all along the inflamed retinal vascular tree. Occlusion of retinal veins may occur at any location from the central retinal vein to the tiny small branches, and it is recurrent [Figure 2a]. After the resolution of retinal hemorrhages, ghost vessels are seen on ophthalmoscopy, and fluorescein angiography may show extensive retinal capillary nonperfusion. Neovascularization of the disc (NVD) or elsewhere (NVE) may develop as a complication of retinal ischemia. However, NVD is more commonly induced by uncontrolled intraocular inflammation. We have reported retinal ischemia in only 13% of eyes with NVD associated with Behçet's uveitis.^24^

Laser flare photometry and fundus fluorescein angiography are the most useful tools in monitoring Behçet's uveitis.^25^–^27^ In between attacks, that is, in the absence of acute inflammatory signs such as anterior chamber cells, retinal infiltrates, or inflammatory sheathing of retinal veins, it may be difficult to determine clinically whether the eye is completely quiet or not. Persistent vitreous haze, hyperemia of the optic disc and blurring of its margins, and macular edema are signs of inadequately controlled intraocular inflammation. Fluorescein angiography is the gold standard to monitor persistent intraocular inflammation which is demonstrated by persistent retinal vascular leakage. It also shows the presence and extent of retinal ischemia [Figure 2b]. Fluorescein angiography is not helpful in eyes with poor visualization of the fundus due to extensive posterior synechiae, cataract, or significant vitreous haze. Anterior chamber flare readings measured by laser flare photometry correlate not only with inflammatory signs in the anterior segment, but also with inflammatory signs in the posterior segment.^25^ Flare readings correlate with fluorescein angiographic leakage in eyes in clinical remission.^25^ We use laser flare photometry on a routine basis as an objective, quantitative and noninvasive measure of intraocular inflammation in Behçet's patients. The risk of a recurrent uveitis attack is higher in patients with flare readings higher than 6 photons/msec than in patients with lower flare readings.^25^

The most common complications of Behçet's uveitis are cataract, posterior synechiae, macular edema, optic atrophy, and glaucoma.^18^,^23^ Retinal neovascularization, retinal tears and detachment, macular hole, and hypotony or phthisis bulbi are uncommon or rare complications. The most common causes of permanent visual loss are maculopathy and optic atrophy. The end-stage fundus picture is characterized by optic atrophy, ghost vessels, diffuse atrophy and gliosis of the retina with variable pigmentation and macular scarring^23^ [Figure 3]. The vitreous is remarkably clear at the end stage.

**Figure 1a:**
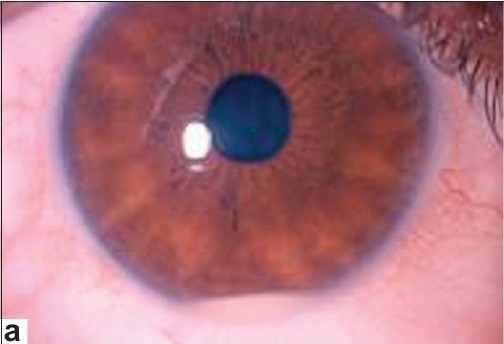
(a) A 27 year-old pregnant woman with Behçet's disease presented with a hypopyon panuveitis;

**Figure 1b:**
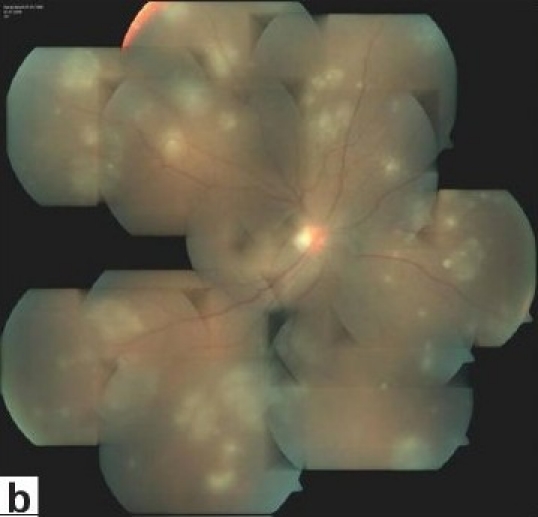
(b)with multifocal retinal infiltrates;

**Figure 1c:**
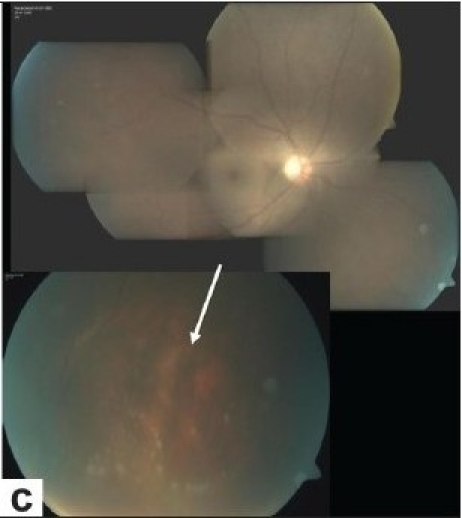
(c)Spontaneous resolution of the retinal infiltrates and accumulation of precipitates on the surface of the inferior peripheral retina were noted one week later

**Figure 2a:**
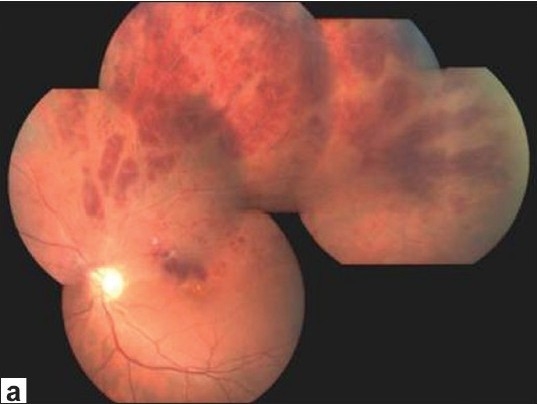
(a)Color fundus photograph of the left eye of a patient with Behçet's disease shows retinal hemorrhages at the posterior pole and at the superior temporal quadrant following an episode of superior temporal branch retinal vein occlusion

**Figure 2b:**
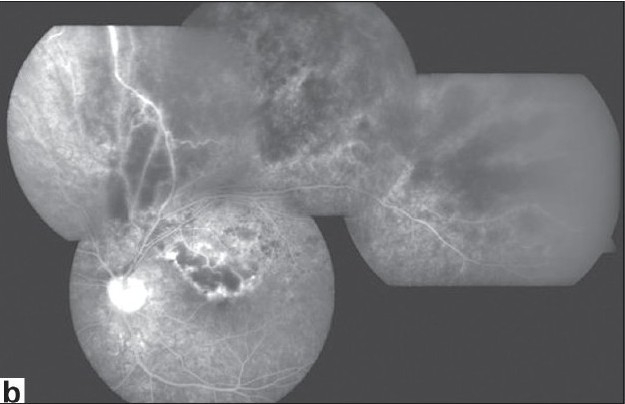
(b) fluorescein angiography shows staining of the optic disc as well as inflamed retinal vessels and hypofluorescence due to retinal hemorrhages and nonperfusion of the retinal vasculature at the involved quadrant

**Figure 3:**
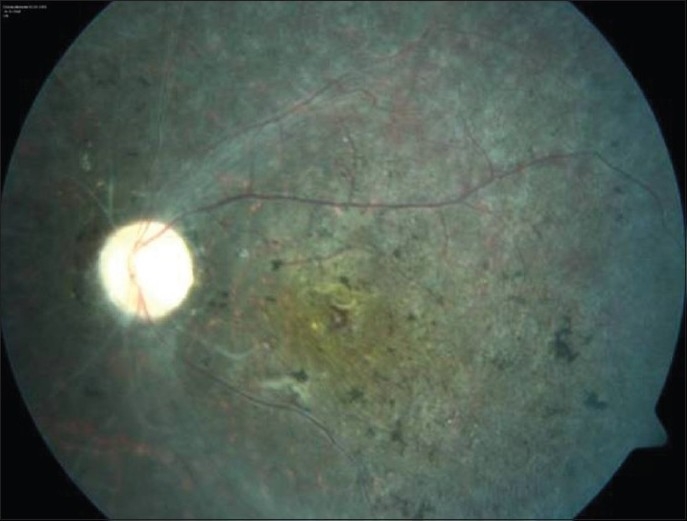
Color photograph shows end-stage fundus picture in the left eye of a patient with Behçet's disease. Please note optic atrophy, gliotic vascular sheathing, ghost vessels, and diffuse retinal atrophy with pigmentation resembling retinitis pigmentosa

## VISUAL PROGNOSIS AND TREATMENT

The frequency and severity of uveitis attacks show individual variability. Although some attacks may cause permanent loss of useful vision, recurrent attacks and cumulative damage usually determine the visual outcome. In general, young males have a more severe disease course and worse visual prognosis.^28^ Other risk factors that have been postulated as indicators of poor visual prognosis include skin lesions, arthritis, neurologic involvement, vascular thrombosis, posterior uveitis attacks, more than three uveitis attacks per year, strong vitreous opacity, exudates within the retinal vascular arcade, and fluorescein angiographic findings of NVD and macular ischemia.^29^–^31^ Recent reports from Japan suggest that improvement in the environment or health care may lead to a change in the epidemiological features of the disease over time in populations with a stable genetic makeup.^32^,^33^ In a study of Japanese patients, Yoshida *et al*. reported a reduction in the frequency of uveitis attacks and in the need for cyclosporine or cyclophosphamide therapy, and an improvement of visual prognosis in the 1990s compared to the 1980s, suggesting a trend to milder disease in the Japanese population.^32^

In a retrospective cohort study conducted in the United States, the rate of loss of visual acuity to 20/200 or worse was 0.09 per eye-year.^34^ In an international retrospective survey, one quarter of the patients had poor vision (< 0.1). 35 Visual outcome was analyzed by the Kaplan-Meier method in two large series reported from Turkey and China.^18^,^23^ The estimated risk of loss of useful vision at 10 years was 30% in males and 17% in females in the Turkish study.^23^ By comparison, these Figures were 65% and 33%, respectively, in the Chinese study.^18^ The authors explained this difference by late referral and noncompliance of Chinese patients with treatment and follow-up visits because of long traveling distances or economic reasons. They stated that visual outcome was better in patients who received early treatment and were compliant with treatment. In the Turkish study, visual outcome was better in patients who presented in the 1990s compared to those who presented in the 1980s.^23^ This was explained by an earlier and more aggressive immunosuppressive treatment of patients in the 1990s. Khairallah *et al*., also reported better final visual acuity in Tunisian patients where immunosuppressive drugs were used as first line therapy after 2001.^36^ In all of these series, only conventional immunosuppressive agents were used.

Behçet's uveitis is one of the absolute indications of immunomodulatory therapy. However, in patients with strictly anterior uveitis, there is no need for systemic treatment. Cells in the posterior vitreous cavity and any leakage on fluorescein angiography should be considered as an indication for immunomodulatory therapy even if there is no other clinical sign in the posterior segment. Systemic corticosteroids should be used only in patients with active inflammation with an immediate threat to vision. A slow tapering of corticosteroids is important because rebound attacks may be more severe than the natural course of the disease. The use of colchicine as an anti-inflammatory agent is limited to mucocutaneous and joint manifestations because it does not have any effect on eye disease. Among the conventional immunomodulatory agents, both azathioprine and cyclosporine have been shown to be effective in controlled trials.^37^,^38^ A combination of these agents may be used when monotherapy fails.^39^ Cyclosporine may be combined with mycophenolate in patients who do not tolerate azathioprine. Cyclosporine is contraindicated in patients with neurological involvement.

The use of alkyllating agents is limited due to their potential serious side effects. Further, cyclosporine has been shown to be superior to intravenous pulsed cyclophosphamide in a comparative study,^40^ and there is no controlled study with chlorambucil. According to the recently published evidence based EULAR recommendations, any Behçet's patient with posterior segment involvement should be treated with azathioprine and corticosteroids, and any patient with severe eye disease should receive either cyclosporine or infliximab infusions combined with corticosteroids and azathioprine; or alternatively interferon alpha therapy.^41^

The introduction of biologic agents into the therapeutic armamentarium has enabled prevention of visual loss in most severe cases of Behçet's uveitis. Although there are no controlled trials yet, remarkable results have been reported with the use of interferon alfa and anti-TNF alfa monoclonal antibody, infliximab, in patients with Behçet's uveitis resistant to conventional treatment.s^42^–^51^ Interferon alfa is effective in over 90% of such cases.^42^–^45^ The main advantage of this agent over other therapeutic regimens seems to be the induction of long-term remissions after discontinuation of treatment.

A single infusion of infliximab 5 mg/kg suppresses the intraocular inflammation rapidly in Behçet's patients. Therefore, it can be used for the treatment of severe attacks with a high risk of structural damage and permanent visual loss.^46^,^47^ However, repeated infusions need to be administered for the prevention of recurrent attacks. Behçet's patients with severe uveitis usually require infusions at intervals shorter than 8 weeks.^48^ Lack of a sustained remission, high cost of treatment, and major safety issues limit the long-term use of infliximab. Furthermore, resistance may develop after prolonged treatment because of the development of antibodies against the murine component of infliximab. A limited number of patients who successfully switched from infliximab to adalimumab, a humanized monoclonal anti-TNF antibody, have been reported in the literature.^52^,^53^

Different health care systems, economic status of different countries, and high cost of biologic agents may limit the physicians' therapeutic choices. For example, infliximab has been approved for the treatment of Behçet's uveitis only in Japan, but still remains as an off-label treatment in other countries. Our stepwise therapeutic approach in Turkish patients is determined by our healthcare system. We use azathioprine and/ or cyclosporine as the initial choice of treatment in patients with posterior segment involvement. Corticosteroids are combined on an individual basis. When the disease is not controlled with the triple-agent regimen that includes azathioprine, cyclosporine, and low-dose steroids or when this regimen is not tolerated, we administer interferon alfa monotherapy. We use infliximab only in patients who fail or do not tolerate interferon alfa therapy. With this approach visual prognosis of our patients has improved in the 2000s compared to the 1990s.

## References

[1] Behçet H (1937). über rezidivierende aphthöse durch ein Virus verursachte Geschwüre am Mund, am Auge und an den Genitalien. Dermatol Wochenschr.

[2] Evereklioglu C (2005). Current concepts in the etiology and treatment of Behçet disease. Surv Ophthalmol.

[3] Verity DH, Marr JE, Ohno S, Wallace GR, Stanford MR (1999). Behçet's disease, the Silk Road and HLA-B51: Historical and geographical perspectives. Tissue Antigens.

[4] Keino H, Okada AA (2007). Behçet's disease: Global epidemiology of an old Silk Road disease. Br J Ophthalmol.

[5] Azizlerli G, Köse AA, Sarica R, Gül A, Tutkun IT, Kulaç M (2003). Prevalence of Behçet's disease in Istanbul, Turkey. Int J Dermatol.

[6] Ohno S, Ohguchi M, Hirose S, Matsuda H, Wakisaka A, Aizawa M (1982). Close association of HLA-Bw51 with Behçet's disease. Arch Ophthalmol.

[7] Gül A (2005). Behçet's disease as an autoinflammatory disorder. Curr Drug Targets Inflamm Allergy.

[8] Direskeneli H (2006). Autoimmunity vs autoinflammation in Behçet's disease: Do we oversimplify a complex disorder?. Rheumatology.

[9] Kitaichi N, Ohno S (2008). Behçet disease in children. Int Ophthalmol Clin.

[10] Tugal-Tutkun I, UrgancIoglu M (2003). Childhood-onset uveitis in Behçet disease: A descriptive study of 36 cases. Am J Ophthalmol.

[11] Saricaoglu H, Karadogan SK, Bayazit N, Yucel A, Dilek K, Tunali S (2006). Clinical features of late-onset Behçet's disease: Report of nine cases. Int J Dermatol.

[12] International Study Group for Behçet's disease (1990). Criteria for diagnosis of Behçet's disease. Lancet.

[13] The International Study Group for Behçet's Disease (1992). Evaluation of diagnostic ('classification') criteria in Behçet's disease-towards internationally agreed criteria. Br J Rheumatol.

[14] Yazici H, Fresko I, Yurdakul S (2007). Behçet's syndrome: Disease manifestations, management, and advances in treatment. Nat Clin Pract Rheumatol.

[15] Yates PA, Michelson JB (2006). Behçet disease. Int Ophthalmol Clin.

[16] Kural-Seyahi E, Fresko I, Seyahi N, Ozyazgan Y, Mat C, Hamuryudan V (2003). The long-term mortality and morbidity of Behçet syndrome: A 2-decade outcome survey of 387 patients followed at a dedicated center. Medicine (Baltimore).

[17] Akman Demir G, Serdaroglu P, Tasci  Bgroup (1999). Neuro-Behçet study Clinical patterns of neurologic involvement in Behçet's disease: Evaluation of 200 patients. Brain.

[18] Yang P, Fang W, Meng Q, Ren Y, Xing L, Kijlstra A (2008). Clinical features of Chinese patients with Behçet's disease. Ophthalmology.

[19] Zamir E, Bodaghi B, Tugal-Tutkun I, See RF, Charlotte F, Wang RC (2003). Conjunctival ulcers in Behçet's disease. Ophthalmology.

[20] Tarzi MD, Lightman S, Longhurst HJ (2005). An exacerbation of Behçet's syndrome presenting with bilateral papillitis. Rheumatology.

[21] Lew H, Lee JB, Han SH, Kim HS, Kim SK (1999). Neuro-Behçet's disease presenting with isolated unilateral lateral recturs muscle palsy. Yonsei Med J.

[22] Hammami S, Yahia SB, Mahjoub S, Khairallah M (2006). Orbital inflammation associated with Behçet's disease. Clin Exp Ophthalmol.

[23] Tugal-Tutkun I, Onal S, Altan-Yaycioglu R, Altunbas HH, Urgancioglu M (2004). Uveitis in Behçet disease: An analysis of 880 patients. Am J Ophthalmol.

[24] Tugal-Tutkun I, Onal S, Altan-Yaycioglu R, Kir N, Urgancioglu M (2006). Neovascularization of the optic disc in Behçet's disease. Jpn J Ophthalmol.

[25] Tugal-Tutkun I, Cingü K, Kir N, Yeniad B, Urgancioglu M, Gül A (2008). Use of laser flare-cell photometry to quantify Intraocular Inflammation in patients with Behçet uveitis. Graefes Arch Clin Exp Ophthalmol.

[26] Yang P, Fang W, Huang X, Zhou H, Wang L, Jiang B (2008 ). Alterations of aqueous flare and cells detected by laser flare-cell photometry in patients with Behçet's disease. Int Ophthalmol in press.

[27] Gedik S, Akova YA, Yilmaz G, Bozbeyoglu S (2005). Indocyanine green and fundus fluorescein angiographic findings in patients with active ocular Behçet's disease. Ocul Immunol Inflamm.

[28] Yazici H, Tüzün Y, Pazarli H, Yurdakul S, Ozyazgan Y, Ozdogan H (1984). Influence of age of onset and patient's sex on the prevalence and severity of manifestations of Behçet's syndrome. Ann Rheum Dis.

[29] Sakamoto M, Akazawa K, Nishioka Y, Sanui H, Inomata H, Nose Y (1995). Prognostic factors of vision in patients with Behçet disease. Ophthalmology.

[30] Demiroglu H, Barista I, Dundar S (1997). Risk factor assessment and prognosis of eye involvement in Behçet's disease in Turkey. Ophthalmology.

[31] Yu HG, Kim MJ, Sewoong F (2009). Fluorescein angiography and visual acuity in active uveitis with Behçet disease. Ocul Immunol Inflamm.

[32] Yoshida A, Kawashima H, Motoyama Y, Shibui H, Kaburaki T, Shimizu K (2004). Comparison of patients with Behçet's disease in the 1980s and 1990s. Ophthalmology.

[33] Wakabayashi T, Morimura Y, Miyamoto Y, Okada AA (2003). Changing patterns of intraocular inflammatory disease in Japan. Ocul Immunol Inflamm.

[34] Kaçmaz RO, Kempen JH, Newcomb C, Gangaputra S, Daniel E, Levy-Clarke GA (2008). Ocular inflammation in Behçet disease: Incidence of ocular complications and of loss of visual acuity. Am J Ophthalmol.

[35] Kitaichi N, Miyazaki A, Iwata D, Ohno S, Stanford MR, Chams H (2007). Ocular features of Behçet's disease: An international collaborative study. Br J Ophthalmol.

[36] Khairallah M, Attia S, Yahia SB, Jenzeri S, Ghrissi R, Jelliti B (2009). Pattern of uveitis in Behçet's disease in a referral center in Tunisia, North Africa. Int Ophthalmol.

[37] Yazici H, Pazarli H, Barnes CG, Tüzün Y, Ozyazgan Y, Silman A (1990). A controlled trial of azathioprine in Behçet's syndrome. N Engl J Med.

[38] Masuda K, Urayama A, Kogure M, Nakajima A, Nakae K, Inaba G (1989). Double-masked trial of cyclosporin versus colchicine and long-term open study of cyclosporin in Behçet's disease. Lancet.

[39] Yazici H, Ozyazgan Y (1999). Medical management of Behçet's syndrome. Dev Ophthalmol.

[40] Ozyazgan Y, Yurdakul S, Yazici H, Tüzün B, Isçimen A, Tüzün Y (1992). Low dose cyclosporin A versus pulsed cyclophosphamide in Behçet's syndrome: A single masked trial. Br J Ophthalmol.

[41] Hatemi G, Silman A, Bang D, Bodaghi B, Chamberlain AM, Gul A (2008). EULAR recommendations for the management of Behçet disease. Ann Rheum Dis.

[42] Kötter I, Zierhut M, Eckstein AK, Vonthein R, Ness T, Günaydin I (2003). Human recombinant interferon alfa-2a for the treatment of Behçet's disease with sight threatening posterior or panuveitis. Br J Ophthalmol.

[43] Tugal-Tutkun I, Güney-Tefekli E, Urgancioglu M (2006). Results of interferon-alfa therapy in patients with Behçet uveitis. Graefes Arch Clin Exp Ophthalmol.

[44] Krause L, Altenburg A, Pleyer U, Köhler AK, Zouboulis CC, Foerster MH (2008). Longterm visual prognosis of patients with ocular Adamantiades-Behçet's disease treated with interferon- alpha-2a. J Rheumatol.

[45] Gueudry J, Wechsler B, Terrada C, Gendron G, Cassoux N, Fardeau C (2008). Long-term efficacy and safety of low-dose interferon alpha2a therapy in severe uveitis associated with Behçet disease. Am J Ophthalmol.

[46] Sfikakis PP, Kaklamanis PH, Elezoglou A, Katsilambros N, Theodossiadis PG, Papaefthimiou S (2004). Infliximab for recurrent, sight-threatening ocular inflammation in Adamantiades-Behçet disease. Ann Intern Med.

[47] Sfikakis PP, Markomichelakis N, Alpsoy E, Assaad-Khalil S, Bodaghi B, Gul A (2007). Anti-TNF therapy in the management of Behçet's disease-review and basis for recommendations. Rheumatology.

[48] Tugal-Tutkun I, Mudun A, Urgancioglu M, Kamali S, Kasapoglu E, Inanc M (2005). Efficacy of infliximab in the treatment of uveitis resistant to the combination of azathioprine, cyclosporine, and corticosteroids in Behçet's disease: An open-label trial. Arthritis Rheum.

[49] Abu El-Asrar AM, Abboud EB, Aldibhi H, Al-Arfaj A (2005). Long-term safety and efficacy of infliximab therapy in refractory uveitis due to Behçet's disease. Int Ophthalmol.

[50] Niccoli L, Nannini C, Benucci M, Chindamo D, Cassarà E, Salvarani C (2007). Long-term efficacy of infliximab in refractory posterior uveitis of Behçet's disease: A 24-month follow-up study. Rheumatology.

[51] Tabbara KF, Al-Hemidan AI (2008). Infliximab effects compared to conventional therapy in the management of retinal vasculitis in Behçet disease. Am J Ophthalmol.

[52] van Laar JM, Missotten T, van Daele PL, Jamnitski A, Baarsma GS, van Hagen PM (2007). Adalimumab: A new modality for Behçet's disease?. Ann Rheum Dis.

[53] Mushtaq B, Saeed T, Situnayake RD, Murray PI (2007). Adalimumab for sight-threatening uveitis in Behçet's disease. Eye.

